# 10 years of Facts Views & Vision in Obgyn: Chief editor's report

**Published:** 2018-12

**Authors:** W Ombelet

“There is a need for a journal that starts off humbly and that can grow by sticking to its founding principle of broad diversity in combination with different levels of intensity. Facts alone tend to be isolated in every narrowing niche of professionalism; views alone tend to degenerate into strong opinions nobody is interested in; and vision alone tends to become a nightmare if unsupported by facts and a beholder’s sharp view. In conjunction, they constitute the basic elements needed for a truly translational medicine, where findings from the bench may become part of a clinician’s view and where today’s clinical expertise may take a jump from a sleepwalker’ s routine to the disruptive insight of the visionary”

Jan Gerris, Editorial, Facts Views Vis Obgyn, Vol 1, Nr 1

“FV&V is a paragon of 21st century communication. In addition to new information, emphasis on discussion and the social interface makes it special while it strives to put related subspecialties in orbit around the star named reproduction.”

Howard Jones, Editorial, Facts Views Vis ObGyn, Vol 3, Nr 2

It’s always nice to look back and try to summarize what we achieved during these last 10 years of FV&V. We observed a broad input from all different fields including obstetrics, perinatal medicine, gynaecology and reproductive health. It was fascinating that many papers showed a lot of interest in the global perspective of our specialty. Structured reviews, strong opinion and viewpoint papers, excellent historical reviews, exactly what we were looking for from the very beginning.

This could only be realized by an outstanding editorial board, a high number of faithful referees, the hard and dedicated work of the ‘Universa Press’ team, the scientific support of our universities and the financial input of the pharmaceutical industry.

Thanks to our artistic reviewer, Koen Vanmechelen, our Journal got a different but very attractive look when compared to other scientific journals. The confrontation of art and science turned out to be unique: “One picture is worth ten thousand words” (Chinese proverb).

In 2014, we succeeded to become PubMed-cited, the process was arduous and complex, but we finally got it. Thes was a major achievement in the history of our Journal. Nowadays the Journal is coverded by major indexing services such as BMJ Clinical Evidence, PMC (PubMed Central), Pubmed, Web of Science, Google Scholar and Europe PMC.

Besides the four regular issues published annually, we also published six Monographs. The first two Monographs on “Artificial Insemination” and “Social aspects of infertility care in developing countries” were published in 2010 and distributed to more than 8000 participants at the annual ESHRE meeting in Rome.

**Figure:**
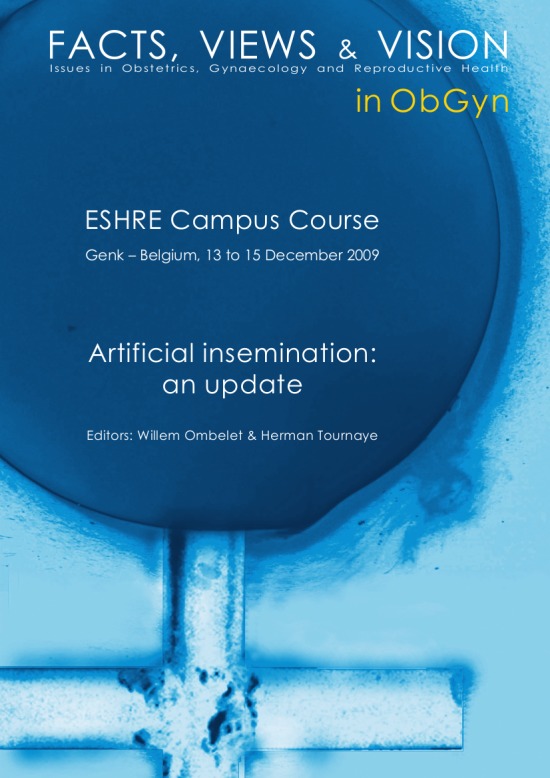


**Figure:**
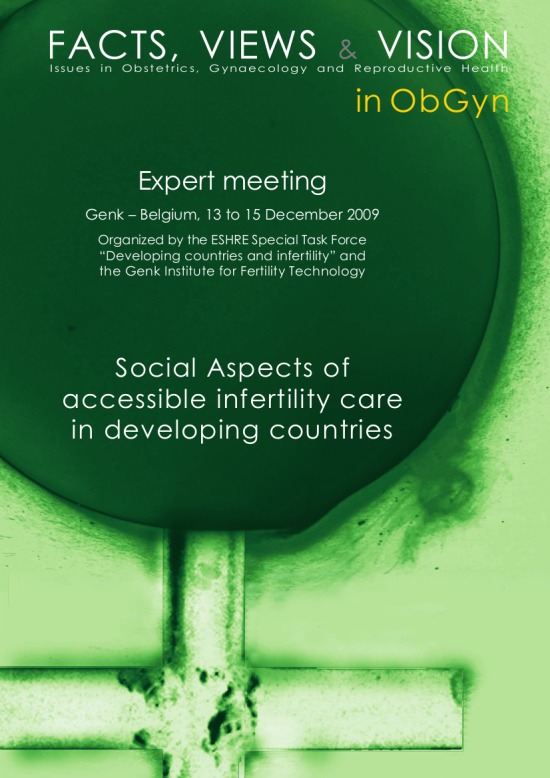


The 3^rd^ Monograph “When training becomes fun for trainers and trainees” was published in 2012 and focused on medical education in obstetrics and gynaecology. It became a very attractive book with 13 papers, written by prominent colleagues from Europe and other parts of the world, describing a number of current issues and developments in training within our field. This Monograph was distributed to all participants of the EBCOG 2012 meeting in Tallinn, Estland.

**Figure:**
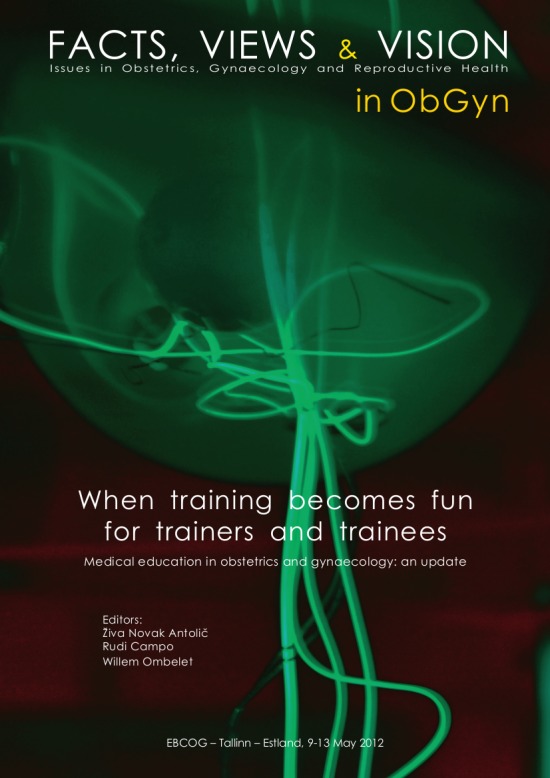


The 4^th^ Monograph on “Biomedical infertility care in poor resources countries: Barriers, Access and Ethics” was also published in 2012 and distributed to more than 10000 participants at the annual ESHRE meeting in Istanbul, Turkey. This was the result of a successful collaboration between the ESHRE Special Task Force on “Developing countries and infertility”, the WHO, the University of Amsterdam and the Walking Egg non-profit organisation.

**Figure:**
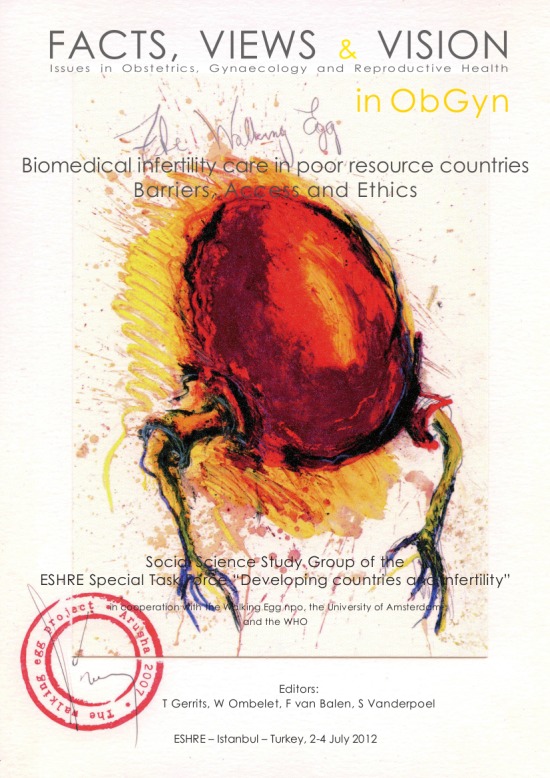


The 5^th^ Monograph “Preterm birth” was edited by Robert E. Garfield, Phoenix, USA, which turned out to become a very attractive book, with 12 papers from around the globe from some of the leading experts on preterm labor and birth. In the USA copies of the monograph were sent to all members of the Society for Maternal-Fetal Medicine and the Society for Gynecological Investigation.

**Figure:**
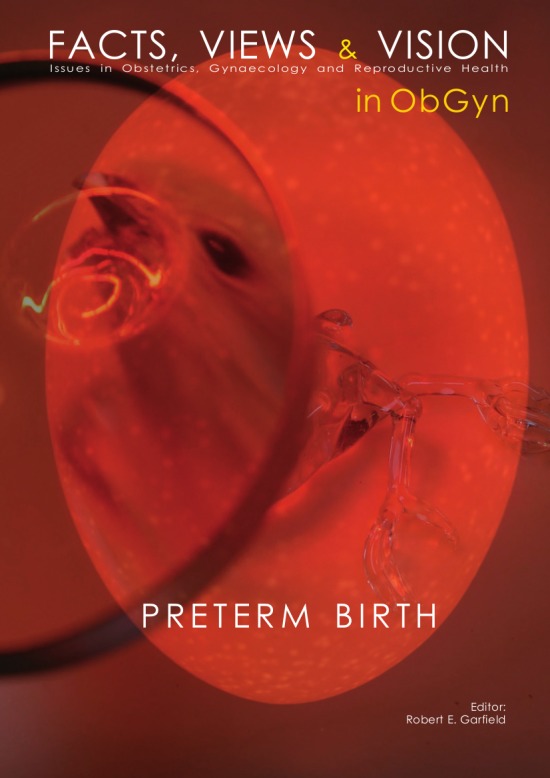


On the occasion of a meeting in Cape Town, South-Africa, we published and distributed our 6th Monograph in bringing together the main actors of the Walking Egg Project and representatives of the interested centres and patient groups in Africa. The final objective of the Walking Egg Project is the implementation of infertility services in many developing countries preferably integrated in existing family planning and mother care services.

## Journal performance

Submissions: Out of 582 submissions, 310 papers were published, not taking into account the 40 editorials, 6 “A tribute to” papers and 12 “History of Medicine” contributions. Table I gives an overview of the different nature of the publications. The acceptance rate turned out to be 53.2 %.

**Table I:** list of different papers published in “Facts Views & Vision in ObGyn (period: 2009-2018)

Original papers	127
Reviews // Structured reviews	46
Perspective, Opinion and Viewpoint papers	66
Summaries of PhDs	22
Short communications	11
Case reports // Case series	22
Editorials	40
EBCOG	3
Letters to the editor	2
A tribute to	6
Who’s Who	11
History of Medicine	12
Images Koen Vanmechelen	76

It was our aim from the beginning to give a forum to “Views and Vision” papers. More than 20% of our publications (66/310) were “Opinion and Viewpoint” papers, and this shows exactly what we were standing for. Although PhD-summaries and Who’s Who papers don’t contribute to a reasonable Impact Factor, we are proud and happy to have published them.

In Table II we report the most cited FV&V publications in the period 2009-2017. We were positively surprised about the impact of our papers. I believe that we have proven that even without being supported by a major scientific society and without substantial funding it is possible not only to survive, but also to have considerable impact on the actual scientific literature of obstetrics, gynaecology and reproductive health.

**Table II:** 25 most frequent cited papers (period: 2009-2017)

Number of citation	Article name and author(s)	Year of publication
76	The social and cultural consequences of being childless in poor-resource areas. *van Balen F, Bos HM. Review.*	2009
67	Incessant ovulation and ovarian cancer - a hypothesis re-visited. *Fathalla MF.*	2013
51	Preterm Cervical Ripening in humans. *Ekman-Ordeberg G, Dubicke A. Review.*	2012
48	Teaching professionalism - Why, What and How. *Cruess SR, Cruess RL.*	2012
43	Global access to infertility care in developing countries: a case of human rights, equity and social justice. *Ombelet W.*	2011
42	Recurrence of endometriosis after hysterectomy. *Rizk B, Fischer AS, Lotfy HA et al. Review.*	2014
40	Trends of Infertility and Childlessness in India: Findings from NFHS Data. *Ganguly S, Unisa S.*	2010
40	The economic impact of infertility on women in developing countries - a systematic review. *Dyer SJ, Patel M. Review.*	2012
38	The first 3,000 Non-Invasive Prenatal Tests (NIPT) with the Harmony test in Belgium and the Netherlands. *Willems PJ, Dierickx H, Vandenakker E et al.*	2014
34	Declining birth rate in Developed Countries: A radical policy re-think is required. *Nargund G.*	2009
34	A short history of sonography in obstetrics and gynaecology. *Campbell S.*	2013
29	Prenatal diagnosis of congenital renal and urinary tract malformations. *Hindryckx A, De Catte L. Review.*	2011
29	Intra-cavitary uterine pathology in women with abnormal uterine bleeding: a prospective study of 1220 women. *Van den Bosch T, Ameye L, Van Schoubroeck D et al.*	2015
29	Biomedical infertility care in sub-Saharan Africa: a social science-review of current practices, experiences and view points. *Gerrits T, Shaw M. Review.*	2010
26	The Walking Egg Project: Universal access to infertility care - from dream to reality. *Ombelet W.*	2013
26	Practical guidance for applying the ADNEX model from the IOTA group to discriminate between different subtypes of adnexal tumors. *Van Calster B, Van Hoorde K, Froyman W et al.*	2015
26	IVM results are comparable and may have advantages over standard IVF. *Ellenbogen A, Shavit T, Shalom-Paz E. Review.*	2014
25	Pelvic Girdle Pain during or after Pregnancy: a review of recent evidence and a clinical care path proposal. *van Balen F, Bos HM. Review.*	2014
25	Obesity and pregnancy, an epidemiological and intervention study from a psychosocial perspective. *Bogaerts A, Devlieger R, Van den Bergh BR, Witters I.*	2014
25	Artificial insemination history: hurdles and milestones. *Ombelet W, Van Robays J.*	2015

From 2019 on, our Journal will be owned by the European Society for Gynaecological Endoscopy (ESGE) in cooporation with the Walking Egg non-profit organization. It will become the official Journal of the ESGE, this means that the major part of the papers will present the surgical aspects of endoscopic imaging and allied techniques. Another part of the Journal will still publish selected papers as we did before.

We have completed 10 exciting years. On behalf of the editorial board and the publisher of “Facts Views Vis ObGyn”. I would like to thank all the authors and referees who contributed to the journal since 2009. It was an honour and a pleasure to be part of this exciting project. A bright and interesting future is waiting for us.

We still count on our referees and our artistic reviewer, Koen Vanmechelen, who is responsible for the very attractive look of our journal. The confrontation of art and science will be continued.

I look forward to a promising future.

Willem Ombelet Editor-in-Chief

